# Low expression of the metabolism-related gene SLC25A21 predicts unfavourable prognosis in patients with acute myeloid leukaemia

**DOI:** 10.3389/fgene.2022.970316

**Published:** 2022-09-30

**Authors:** Wenjun Wang, Qian Liang, Jingyu Zhao, Hong Pan, Zhen Gao, Liwei Fang, Yuan Zhou, Jun Shi

**Affiliations:** Regenerative Medicine Clinic, State Key Laboratory of Experimental Hematology, National Clinical Research Center for Blood Diseases, Haihe Laboratory of Cell Ecosystem, Institute of Hematology and Blood Diseases Hospital, Chinese Academy of Medical Sciences and Peking Union Medical College, Tianjin, China

**Keywords:** SLC25A21, prognosis, bioinformatics, GEO, TCGA, immune checkpoint, drug sensitivity, acute myeloid leukaemia (AML)

## Abstract

Acute myeloid leukaemia (AML) is a heterogeneous disease associated with poor outcomes. To identify AML-specific genes with prognostic value, we analysed transcriptome and clinical information from The Cancer Genome Atlas (TCGA) database, Gene Expression Omnibus (GEO) datasets, and Genotype-Tissue Expression (GTEx) project. The metabolism-related gene, *SLC25A21* was found to be significantly downregulated in AML, and was associated with high white blood cell (WBC) counts, high pretrial blood (PB) and bone marrow (BM) blast abundance, *FLT3* mutation, *NPM1* mutation, and death events (all *p* value <0.05). We validated the expression of *SLC25A21* in our clinical cohort, and found that *SLC25A21* was downregulated in AML. Moreover, we identified low expression of *SLC25A21* as an independent prognostic factor by univariate Cox regression (hazard ratio [HR]: 0.550; 95% Confidence interval [CI]: 0.358–0.845; *p* value = 0.006) and multivariate Cox regression analysis (HR: 0.341; 95% CI: 0.209–0.557; *p* value <0.05). A survival prediction nomogram was established with a C-index of 0.735, which indicated reliable prognostic prediction. Subsequently, based on the median *SLC25A21* expression level, patients in the TCGA-LAML cohort were divided into low- and high-expression groups. Gene ontology (GO) function and Kyoto Encyclopedia of Genes and Genomes (KEGG) pathway enrichment analyses of DEGs highlighted growth factor binding, extracellular structure organization, cytokine‒cytokine receptor interaction, etc. The results of gene set enrichment analysis (GSEA) indicated that the epithelial-mesenchymal transition, KRAS signalling, oxidative phosphorylation, and reactive oxygen species pathways were enriched. Through gene coexpression and protein‒protein interaction (PPI) network analysis, we identified two hub genes, *EGFR* and *COL1A2*, which were linked to worse clinical outcomes. Furthermore, we found that lower *SLC25A21* expression was closely associated with a significant reduction in the levels of infiltrating immune cells, which might be associated with immune escape of AML cells. A similar trend was observed for the expression of checkpoint genes (*CTLA4*, *LAG3*, *TIGIT*, and *HAVCR2*). Finally, drug sensitivity testing suggested that the low-expression *SLC25A21* group is sensitive to doxorubicin, mitomycin C, linifanib but resistant to JQ1, belinostat, and dasatinib. Hence, our study demonstrated that a low expression level of *SLC25A21* predicts an unfavourable prognosis in patients with AML.

## Introduction

Acute myeloid leukaemia (AML) is a genetically and clinically heterogeneous disease characterized by clonal expansion, differentiation arrest, and evasion of apoptosis. Despite recent advances in chemotherapy, immunotherapy, and bone marrow transplantation, large numbers of AML patients still have a dismal prognosis, with a 5-years survival rate of only approximately 20% (Chen et al., 2019). The development of personalized biomarker-targeted therapies in AML has improved the efficacy of systemic therapies and prolonged patient survival to some extent. However, the lack of biomarkers hinders further improvements in accurate diagnosis and prediction of efficacy. Thus, it is extremely important to discover novel diagnostic and prognostic biomarkers for targeted therapy in AML.

In this research, various comprehensive bioinformatics and statistical methods were used to explore independent prognostic factors in AML. Differentially expressed gene (DEG) analysis, Kaplan-Meier analysis and Cox regression analysis helped us screen out Solute Carrier Family 25 Member 21 (*SLC25A21*) as an AML-specific prognostic marker. *SLC25A21*, also called *ODC*, is a metabolism-related gene located on chromosome 14q13.3, and it encodes a protein known as mitochondrial 2-oxodicarboxylate carrier (Fiermonte et al., 2001). The SLC25A21 protein not only facilitates the counterexchange of the oxodicarboxylates 2-oxoadipate and 2-oxoglutarate but also plays an essential role in the metabolism of several amino acids (Fiermonte et al., 1998; Kunji et al., 2020). Germline *SLC25A21* deficiency in humans causes the depletion of mitochondrial DNA and spinal muscular atrophy-like disease (Fiermonte et al., 1998; Boczonadi et al., 2018). Metabolic reprogramming is a hallmark of cancer, and targeting metabolic factors is an emerging therapeutic modality (Chen et al., 2020a; Bosc et al., 2020; Forte et al., 2020; Pei et al., 2020). Interestingly, a recent study showed that *SLC25A21* is a key tumor suppressor gene in bladder cancer (Wang et al., 2021). However, the potential role of *SLC25A21* in AML and whether it could serve as a novel target for metabolic therapy remain completely unknown.

Hence, we used GO and KEGG analyses, GSEA, PPI network construction, immune infiltration and immune checkpoint correlations, and drug sensitivity analysis to explore the underlying molecular pathological mechanisms of *SLC25A21* in AML. Based on the above results, we confirmed the prognostic value of *SLC25A21* and identified it as a potential therapeutic target for AML.

## Material and methods

### Data source

We included 804 samples from three independent cohorts in this study: the TCGA LAML cohort (RNA-seq, *n* = 132) (Cancer Genome Atlas Research et al., 2013), the GSE13159 microarray dataset (*n* = 573) (Haferlach et al., 2010) and the GSE12417 dataset (RNA-seq, *n* = 163) (Metzeler et al., 2008). The matrix of mRNA expression in normal samples (*n* = 70) was extracted from the GTEx project (Consortium, 2020). The RNA-seq and clinical information from the TCGA LAML and GTEx datasets were acquired using the UCSC XENA browser (https://xenabrowser.net/datapages/) (Vivian et al., 2017; Consortium, 2020; Goldman et al., 2020). The microarray dataset GSE13159 and RNA-seq dataset GSE12417 were downloaded from the GEO database (https://www.ncbi.nlm.nih.gov/geo/). To maintain the comparability of data from different databases, TPM values from RNA-Seq were determined for intrasample comparison after log2 transformation. In our study, specimens with no survival data were excluded.

### Gene expression profiling

To analyse the gene expression profiles of AML, 705 bone samples from the GSE13159 and LAML datasets were used. The GSE13159 dataset was collected from the Microarray Innovations in Leukaemia Study. The DEGs were predicted using the limma package in R, with an adjusted *p* value <0.05 and |log2FC| ≥ 0.15 (Ritchie et al., 2015). A list of 14 common differentially expressed AML-specific genes was obtained from the above databases by using the Venn online tool (https://bioinfogp.cnb.csic.es/tools/venny/).

### Identification of overall survival-related genes

The LAML cohort was used to investigate the potential prognostic significance of the selected genes in AML patients. OS-related genes with a *p* value <0.05 were selected using univariate Cox hazard regression analysis for further research. The external cohort GSE12417 (*n* = 163) was used to validate our results (Metzeler et al., 2008).

### Human subjects and quantitative real-time PCR

Bone marrow samples were collected from 20 patients with AML diagnosis according to the 2016 WHO criteria. We also collected 10 bone marrow samples from healthy donors. The individuals in both cohorts were aged 18–70 years. All patients signed the informed consent form, and the study protocol was approved by the Ethics Committee of our hospital. The patient information collected is listed in Supplementary Table S3. Isolation of mononuclear cells was performed using standard Ficoll standard procedure. Total RNA was isolated with TRIzol reagent (Life Technologies) and then reverse transcribed to cDNA using the ImProm-II™ Reverse Transcription Kit (Promega, Madison, United States). For gene expression analysis, cDNA samples were mixed with SYBR reagent using a 7900 real-time PCR system (Applied Biosystems), and the data were normalized to GAPDH. The primer sequences are available in Supplementary Table S4.

### Differentially expressed genes analysis

Based on the median *SLC25A21* expression level, patients in the LAML cohort were divided into two groups of low and high expression. A list of DEGs was obtained using DESeq2 with an adjusted *p* value < 0.05 and |log2FC| ≥ 1 (Love et al., 2014).

### Gene ontology and kyoto encyclopedia of genes and genomes enrichment analysis of differentially expressed genes

By using the R package clusterProfiler, we carried out functional annotation analysis to investigate the underlying functions of DEGs in AML (Yu et al., 2012; Gene Ontology, 2021; Kanehisa et al., 2021). A Benjamin–Hochberg adjusted *p* value <0.05 was interpreted as statistically significant. Heatmap of clustered DEGs was generated using ClustVis software (Metsalu and Vilo, 2015).

### Gene set enrichment analysis

GSEA was conducted by using the clusterProfiler package in R and hallmark signatures (h.all.v7.2. symbols.gmt) from MsigDB (Subramanian et al., 2005; Yu et al., 2012). Results were considered significant when |NES | >1, normalized *p* value <0.05^23^.

### Comprehensive protein‒protein interaction analysis

Associations between *SLC25A21* and the expression of other genes were assessed using the LinkedOmics database (http://www.linkedomics.org/login.php). We derived the PPI network from the STRING database (https://string-db.org/) to estimate the interactional correlations of the DEGs (Szklarczyk et al., 2019). A confidence score >0.4 was considered significant. Hub proteins and key nodes in the constructed PPI network were identified using the Cytoscape plugin CytoHubba (Shannon et al., 2003). We investigated the association of *SLC25A21* expression with hub genes through correlation heatmaps by using the R package ggplot2.

### Immune infiltrate analysis

By using Single Sample GSEA (ssGSEA) in the R package gsva, Spearman correlation coefficients were computed between the expression level of *SLC25A21* and ssGSEA-based immune cell infiltration levels (Bindea et al., 2013; Hanzelmann et al., 2013). The involved immune cells were of 22 immune cell subtypes, including B cells, monocytes, macrophages, neutrophils, NK cells, DC cells and all subtypes of T-cells. The relationships between *SLC25A21* expression and the expression of immune checkpoint molecules, including *PDCD1*, *CD274*, *CTLA-4*, *LAG-3*, *TIGIT*, and *HAVCR2*, were identified through correlation heatmaps by using the R package ggplot2.

### Drug sensitivity prediction

The half-maximal inhibitory concentration (IC50), calculated using the pRRophetic package in R (Geeleher et al., 2014; Ding et al., 2021), was used for drug sensitivity prediction.

### Survival and statistical analysis

**FIGURE 1:**
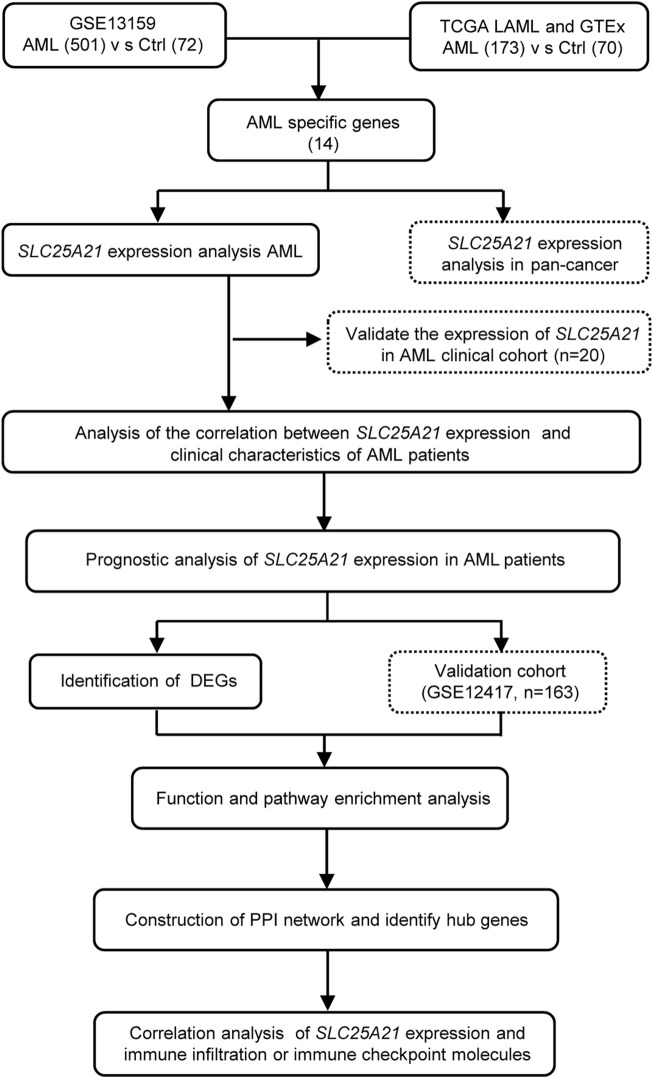
Flow chart of the study.

All statistical analyses were performed with R, version 4.1.3 (https://www.r-project.org/). The Wilcoxon rank-sum test and Kruskal–Wallis test were used to detect differences among continuous variables. The correlation of clinical features with low and high *SLC25A21* expression were analysed with Pearson’s correlation χ2 test. For survival analysis, Cox proportional hazards analysis was conducted by using “survival” and “survminer” in R. Variables significant in Cox univariate analysis were selected for multivariate analysis. The Kaplan-Meier method was used for univariate analyses of OS. Receiver operating characteristic (ROC) curves and AUC values were generated by pROC in R to assess the diagnostic efficacy of *SLC25A21* for AML. All tests were two-sided, and a *p* value < 0.05 was considered to indicate statistical significance. A flow chart of the analyses is presented in Figure 1.

## Results

### Acute myeloid leukaemia-specific genes identified with screening datasets

**FIGURE 2:**
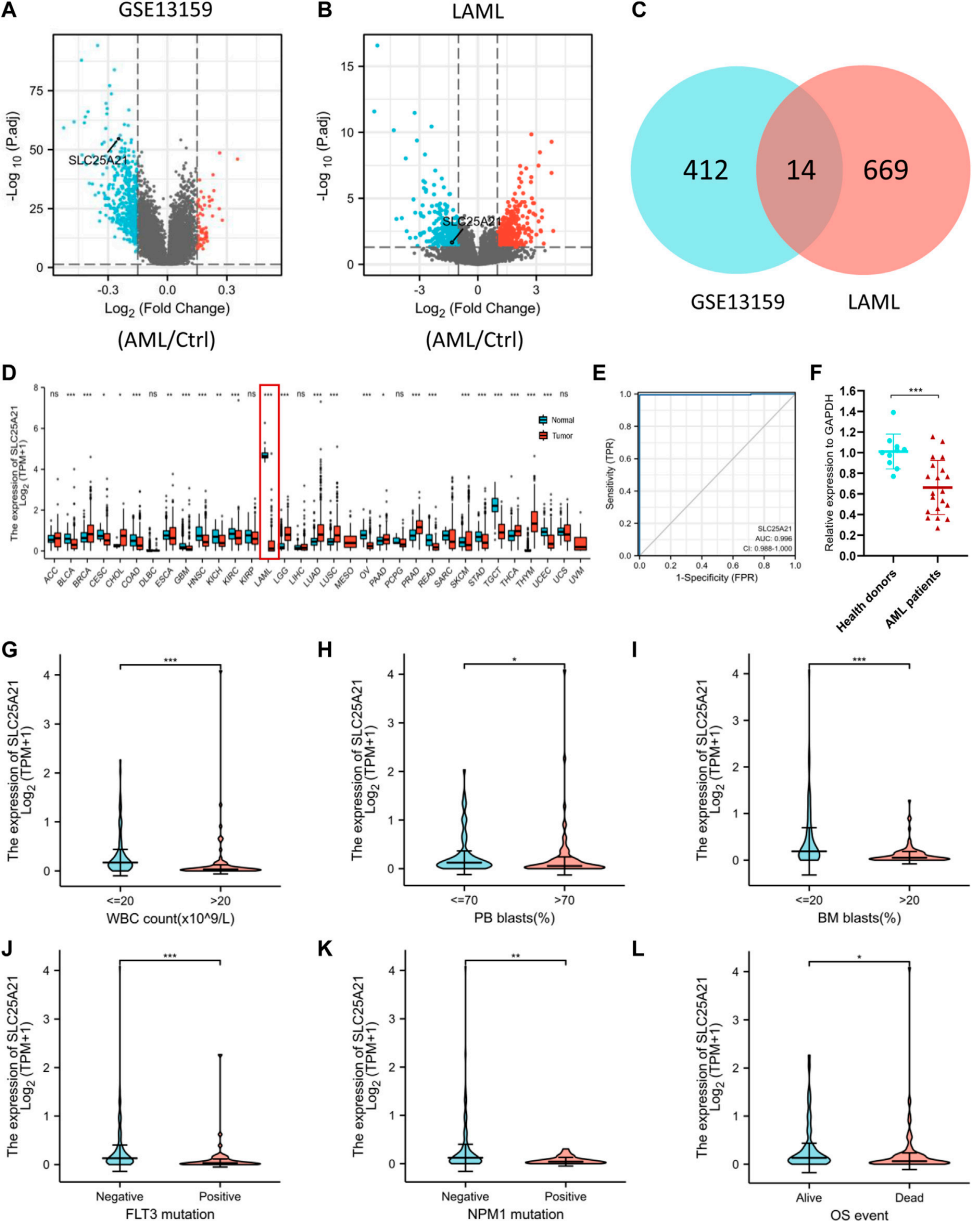
Identifying AML-specific genes and the association of SLC25A21 expression with clinical characteristics. **(A)** Volcano plot displaying DEGs between AML and control bone marrow samples in the GSE13159 dataset. Each point represents the average value of one transcript. **(B)** Volcano plot of DEGs between AML and normal samples in the TCGA-LAML and GTEx datasets. **(C)** Venn diagram of differential gene expression. Selected genes for further analysis based on the intersections of DEGs. **(D)** The level of SLC25A21 expression in different tumor from TCGA and GTEx database. **(E)** Receiver operating characteristic (ROC) analysis of SLC25A21 in AML. The analysis was performed with the TCGA-LAML and GTEx dataset. **(F)** Differential expression of SLC25A21 between AML patients and healthy donors by qPCR analyses. The results were expressed as the fold change of AML patients relative to healthy donors. Clinical characteristics included **(G)** WBC count, **(H)** PB blasts abundance, **(I)** BM blasts abundance, **(J)** FLT3 mutation, **(K)** NPM1 mutation, **(L)** OS evens. Data are presented as the mean ± SD, and represent triplicate wells from one of two independent experiments. *p < 0.05, **p < 0.01, ***p < 0.001. Analysis between two groups of unpaired samples: Wilcoxon rank-sum test, analysis among multiple groups: Kruskal‒Wallis test (ns *p* ≥ 0.05, *p < 0.05, **p < 0.01, ***p < 0.001).

We analysed the TCGA LAML database and the GEO dataset GSE13159 to understand the potential molecular changes in AML. For GSE13159, gene expression analysis was performed on bone marrow samples from 501 AML to 72 control samples (nonleukaemia and healthy donors). We identified 61 upregulated and 365 downregulated DEGs in the AML group (|log2FC| ≥ 0.15, adjusted *p* value < 0.05) by using the limma package in R. The volcano plots are shown in Figure 2A. By screening the TCGA LAML datasets (|log2FC| ≥ 1, adjusted *p* value < 0.05) with DESeq2, a total of 683 differentially expressed genes were obtained, which are shown in Figure 2B. Venn diagram software was used to obtain a common DEG list. A list of 14 intersectional genes was extracted, of which 11 were upregulated and 3 were downregulated in GSE13159 and TCGA LAML (Figure 2C), including *IL1R2*, *MMP8*, *FGF13*, *SLC25A21*, etc. In pan-cancer analysis, we determined the expression profiles of these genes in normal and malignant samples. Remarkably, *SLC25A21* was downregulated in multiple malignancies, especially in AML (Figure 2D). Furthermore, we performed receiver operating characteristic (ROC) curve analyses, and the area under the curve (AUC) was used to evaluate the discriminatory capacity. The calculated AUC value was 0.996 (95% confidence interval, CI = 0.988–1.0, Figure 2E), which means that *SLC25A21 has* excellent discrimination power to distinguish AML patients from normal controls. Finally, we validated the expression of *SLC25A21* in AML patient bone marrow samples collected in our centre. We compared the mRNA expression level of *SLC25A21* between AML patients (*n* = 20) and healthy donors (*n* = 10) by qPCR. *SLC25A21* was significantly downregulated in AML samples, with a *p* value of 0.0007 (Figure 2F). Therefore, *SLC25A21* could be a specific factor to distinguish AML from normal samples.

### Low levels of SLC25A21 are associated with adverse clinical features in acute myeloid leukaemia

To investigate the clinical significance of *SLC25A21*, we analysed the TCGA LAML cohort, which includes 132 AML patients with clinical information. As shown in Figures 2F–K, low *SLC25A21* expression was associated with higher WBC counts (*p* value ＜0.001, Figure 2G), higher PB blast abundance (*p* value ＜0.05, Figure 2H), higher BM blast abundance (*p* value ＜ 0.001, Figure 2I), FLT3 mutation (*p* value ＜ 0.001, Figure 2J), NPM1 mutation (*p* value ＜ 0.01, Figure 2K), and death evens (*p* value ＜ 0.001, Figure 2L); however, no association was found with cytogenetic risk or French–American–British (FAB) classifications (Supplementary Figures 1A–B). In addition, similar trends were observed when patients were grouped by low or high *SLC25A21* expression; more details are shown in Table 1.

**TABLE 1:** Clinical characteristics of AML patients with differential SLC25A21 expression levels.

Characteristics	Total(N)	HR (95%CI) univariate analysis	*p* value univariate analysis	HR (95%CI) multivariate analysis	*p* value multivariate analysis
Gender	140				
Female	63	References			
Male	77	1.030 (0.674–1.572)	0.892		
Age	140				
≤60	79	References			
>60	61	3.333 (2.164–5.134)	<0.001	3.903 (2.425–6.281)	<0.001
WBC count (x10^9/L)	139				
≤20	75	References			
>20	64	1.161 (0.760–1.772)	0.490		
PB blasts (%)	140				
≤70	66	References			
>70	74	1.230 (0.806–1.878)	0.338		
BM blasts (%)	140				
≤20	59	References			
>20	81	1.165 (0.758–1.790)	0.486		
Cytogenetic risk	138				
Favorable	31	References			
Intermediate	76	2.957 (1.498–5.836)	0.002	2.268 (1.135–4.533)	0.020
Poor	31	4.157 (1.944–8.893)	<0.001	3.540 (1.607–7.800)	0.002
FLT3 mutation	136				
Negative	97	References			
Positive	39	1.271 (0.801–2.016)	0.309		
NPM1 mutation	139				
Negative	106	References			
Positive	33	1.137 (0.706–1.832)	0.596		
RAS mutation	139				
Negative	131	References			
Positive	8	0.643 (0.235–1.760)	0.390		
IDH1 R132 mutation	138				
Negative	126	References			
Positive	12	0.588 (0.238–1.452)	0.249		
IDH1 R140 mutation	138				
Negative	127	References			
Positive	11	1.131 (0.565–2.264)	0.727		
IDH1 R172 mutation	138				
Negative	136	References			
Positive	2	0.610 (0.085–4.385)	0.623		
SLC25A21	140				
Low	66	References			
High	74	0.550 (0.358–0.845)	0.006	0.341 (0.209–0.557)	<0.001

### Low expression of *SLC25A21* predicts unfavourable prognosis in patients with acute myeloid leukaemia

**FIGURE 3:**
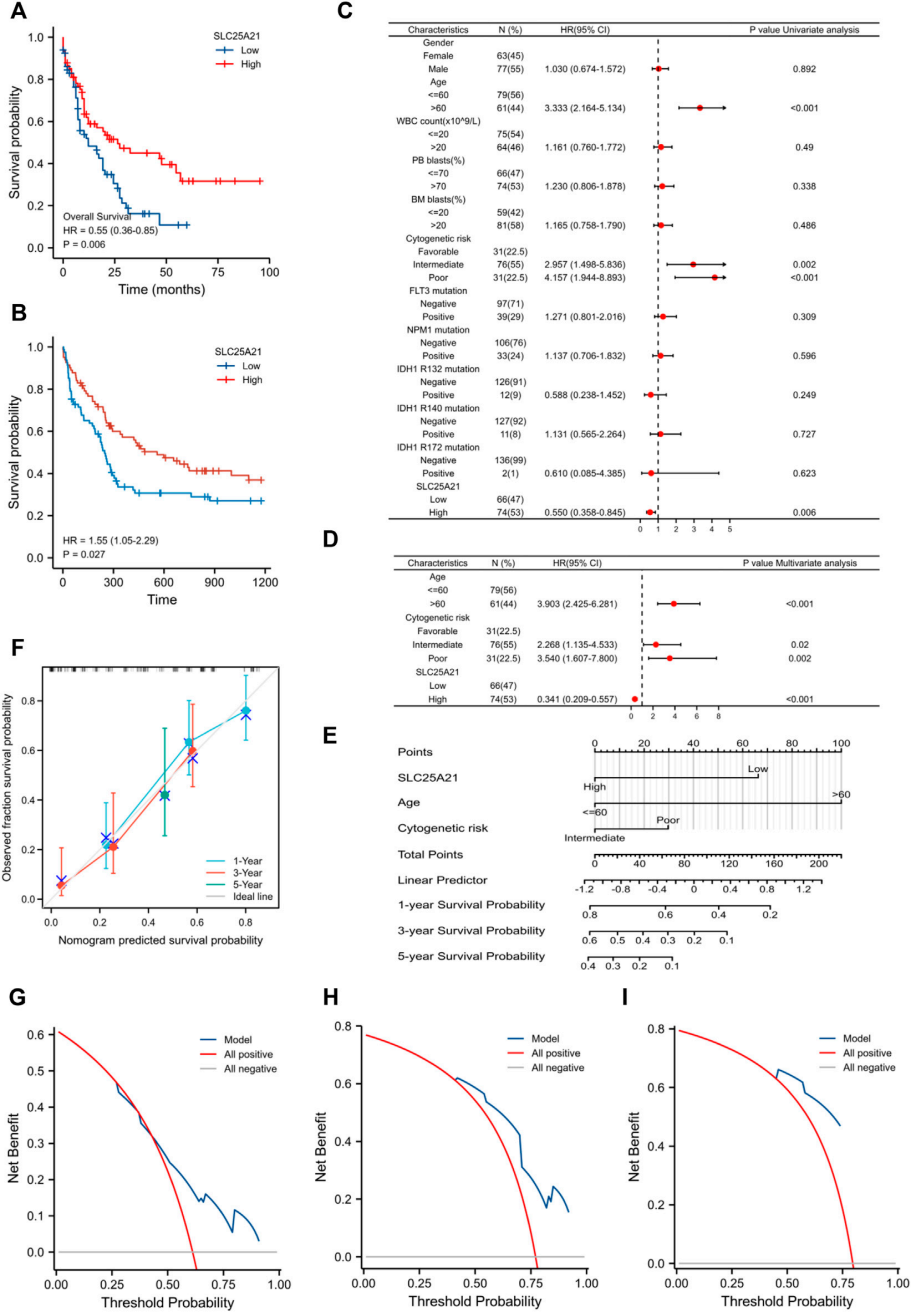
The prognostic value of SLC25A21 in AML. **(A)** Kaplan-Meier curve analysis of overall survival (OS) between the high- and low- SLC25A21 expression groups in the TCGA-LAML dataset. **(B)** OS analysis of SLC25A21 in the independent validation cohort GSE12417. **(C)** Univariate analyses of OS shown as a by forest plot. **(D)** Multivariate analyses of OS shown as a forest plot. **(E)** A nomogram integrating SLC25A21 and other prognostic factors for AML (mut: mutation, wt, wild type; Int, Intermediate; Fav, Favourable). **(F)** The calibration curve of the nomogram. The DCA curves of the nomogram at 1 year **(G)**, 3 years **(H)**, and 5 years **(I)**.

We further investigated the prognostic value of *SLC25A21* in AML. First, patients in the low *SLC25A21* expression group had shorter OS than those in the high *SLC25A21* expression group (*p* value = 0.006, Figure 3A), which indicated that a low *SLC25A21* expression level was associated with an unfavourable prognosis in patients with AML. Furthermore, we validated our results in an independent external validation cohort GSE12417 (*p* value = 0.027, Figure 3B).

In addition, univariate and multivariate logistic regression analyses were performed to determine whether low expression of *SLC25A21* was an independent prognostic factor for AML. Univariate Cox regression analysis showed that low levels of *SLC25A21* expression were associated with poor OS (hazard ratio, [HR]: 0.55; 95% confidence interval [CI]: 0.358–0.845; *p* value = 0.006). Meanwhile, increasing age and unfavourable cytogenetics were also risk factors associated with poor outcomes. Then, all variables significant in univariate Cox regression analysis (*p* value <0.05) were included in multivariate Cox regression analysis. Subsequently, age, unfavourable cytogenetics and low levels of *SLC25A21* expression (HR: 1.733; 95% CI: 1.079–2.781; *p* value = 0.023) were identified as independent prognostic factors for OS. The forest plots present the Cox regression results in Figures 3C,D (more details are provided in Supplementary Table S1).

Moreover, a nomogram including the prediction model was established based on multivariable logistic regression analysis. The established nomogram was well calibrated and had good discriminative power, with a concordance index (C-index) of 0.735 for OS prediction (Figure 3E). Furthermore, we utilized calibration curves and decision curve analysis (DCA) to report the clinical net benefit of our model. The calibration curve at 1, 3, or 5 years still showed high consistency between the predicted survival probability and actual OS proportions (Figure 3F). In addition, the decision curve analysis for the individualized prediction nomogram is presented in Figures 3G–I. In summary, the nomogram model we established had good predictive accuracy for AML patient survival.

### Biological function enrichment of the *SLC25A21* gene in acute myeloid leukaemia

**FIGURE 4:**
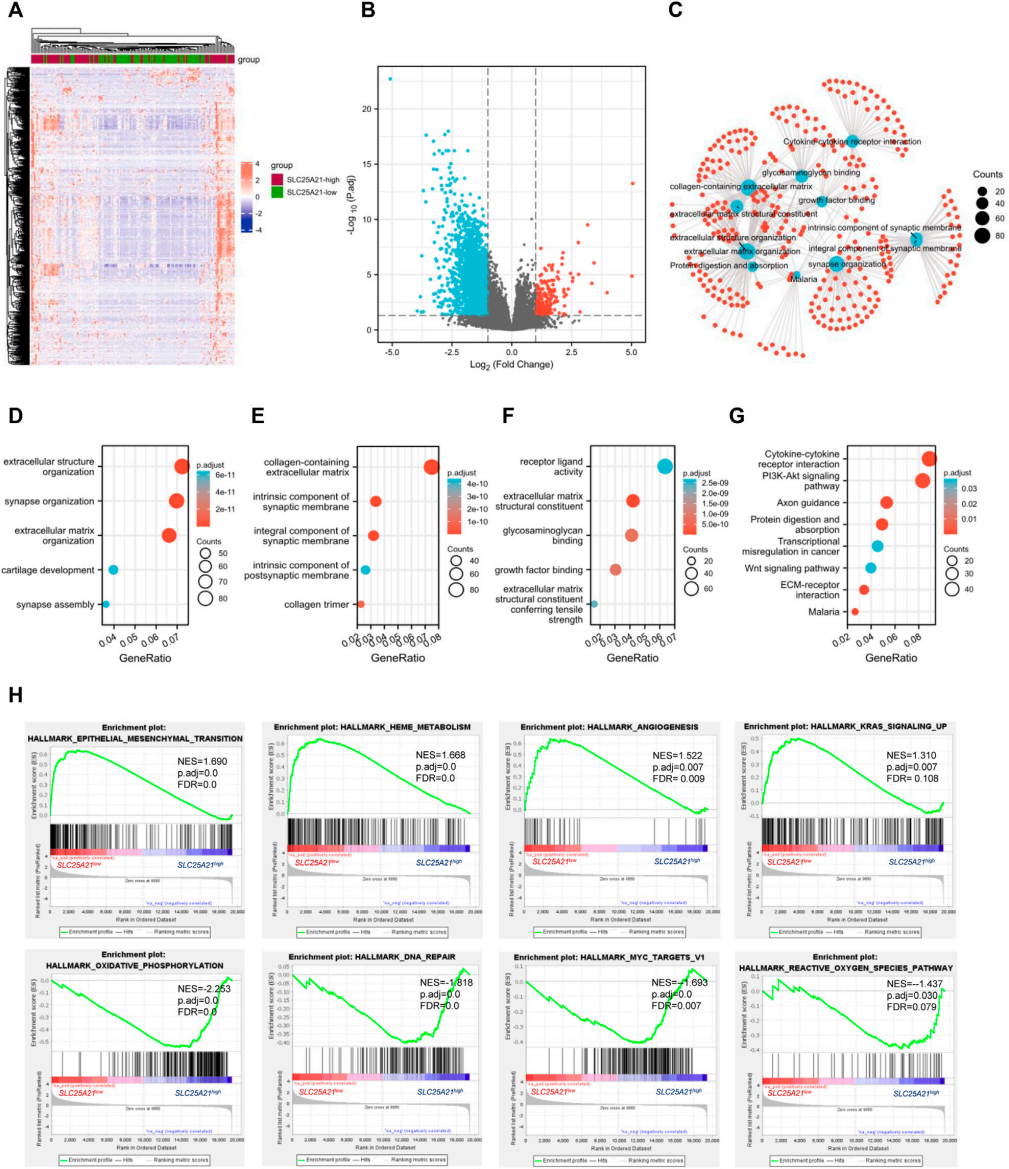
DEGs and functional enrichment of the high- and low- SLC25A21 expression groups in AML. **(A)** Heatmap of SLC25A21-related DEGs. **(B)** Volcano plot of SLC25A21-related DEGs. **(B,C)** Interactive analyses of GO and KEGG, including biological processes **(D)**, cellular components **(E)**, and molecular functions **(F)**, and KEGG pathways of SLC25A21-related DEGs. **(G)** Each red plot in the graph represents a specific gene included in the gene-set. Each blue plot represents the enriched gene-sets. The size of the blue plot represents the number of gene read counts in the gene-sets. **(H)** GSEA of SLC25A21-related DEGs.

Next, we aimed to further investigate the underlying mechanisms and functional pathways of *SLC25A21* in AML. We identified DEGs between the low- and high- *SLC25A21* expression groups. The final list of DEGs included 1,270 genes, with 128 genes upregulated and 1,142 genes downregulated (|log2FC| ≥ 1, adjusted *p* value <0.05). The heatmap and the volcano map are shown in Figures 4A,B.

To elucidate the potential biological function of *SLC25A21* in AML, we performed enrichment analyses. The top 15 GO enrichment items (Figures 4D–F) and top 5 KEGG pathways are shown in Figure 4G. The main enriched GO terms of the DEGs were extracellular structure organization, synapse organization, extracellular matrix organization, collagen-containing extracellular matrix, integral component of synaptic membrane, receptor ligand activity, extracellular matrix structural constituent, glycosaminoglycan binding, growth factor binding, etc.

We found that the enriched pathways included cytokine‒cytokine receptor interaction, PI3K-Akt signalling pathway, focal adhesion, proteoglycans in cancer, transcriptional misregulation in cancer, Wnt signalling pathway, and TGF-beta signalling pathway (Supplementary Table S2). Furthermore, interaction analysis was carried out with the results of GO and KEGG analysis to explore interrelationships. The number of enriched genes was ranked from most to least common: collagen-containing extracellular matrix, extracellular structure organization, extracellular matrix organization, cytokine‒cytokine receptor interaction, extracellular matrix structural constituent, growth factor binding, protein digestion and absorption, malaria (Figure 4C).

Finally, we utilized GSEA to assess key regulatory pathways for *SLC25A21* expression. We found 17 significant pathways associated with *SLC25A21* (Table 2), of which the major affected pathways included epithelial-mesenchymal transition, heme metabolism, angiogenesis, KRAS signalling, oxidative phosphorylation, DNA repair, MYC targets and reactive oxygen species (Figure 4H).

**TABLE 2:** Seventeen items from gene set enrichment analysis. (A) Gene sets enriched in phenotype SLC25A21 low. (B) Gene sets enriched in phenotypeSLC25A21 high.

	GS	SIZE	ES	NES	NOM p-value	FDR q-value	FWER p-value	RANK AT MAX	LEADING EDGE
1	HALLMARK_EPITHELIAL_MESENCHYMAL_TRANSITION	200	0.64	1.69	0	0	0	2921	tags = 45%, list = 15%, signal = 52%
2	HALLMARK_HEME_METABOLISM	197	0.64	1.67	0	0	0	3583	tags = 38%, list = 18%, signal = 46%
3	HALLMARK_ANGIOGENESIS	36	0.64	1.52	0.007	0.01	0.037	2740	tags = 53%, list = 14%, signal = 61%
4	HALLMARK_HEDGEHOG_SIGNALING	36	0.62	1.46	0.02	0.026	0.13	3664	tags = 50%, list = 19%, signal = 62%
5	HALLMARK_ESTROGEN_RESPONSE_EARLY	199	0.55	1.45	0	0.025	0.148	3940	tags = 38%, list = 20%, signal = 47%
6	HALLMARK_APICAL_SURFACE	44	0.6	1.44	0.009	0.023	0.172	3050	tags = 34%, list = 16%, signal = 40%
7	HALLMARK_MYOGENESIS	199	0.52	1.37	0.002	0.056	0.404	2994	tags = 30%, list = 15%, signal = 35%
8	HALLMARK_UV_RESPONSE_DN	144	0.51	1.32	0.012	0.099	0.651	4417	tags = 35%, list = 23%, signal = 45%
9	HALLMARK_KRAS_SIGNALING_UP	199	0.5	1.31	0.007	0.108	0.716	3932	tags = 36%, list = 20%, signal = 45%
11	HALLMARK_SPERMATOGENESIS	134	0.49	1.27	0.037	0.145	0.874	4396	tags = 34%, list = 23%, signal = 43%
12	HALLMARK_ESTROGEN_RESPONSE_LATE	198	0.48	1.26	0.025	0.16	0.917	4280	tags = 36%, list = 22%, signal = 46%
13	HALLMARK_KRAS_SIGNALING_DN	199	0.48	1.25	0.026	0.161	0.934	3498	tags = 33%, list = 18%, signal = 39%

	GS	SIZE	ES	NES	NOM p-value	FDR q-value	FWER p-value	RANK AT MAX	LEADING EDGE
1	HALLMARK_DNA_REPAIR	148	−0.4018086	−1.818951	0	0	0	7204	tags = 64%, list = 37%, signal = 101%
2	HALLMARK_REACTIVE_OXYGEN_SPECIES_PATHWAY	49	−0.3934737	−1.4372096	0.030303031	0.07993332	0.025	4629	tags = 49%, list = 24%, signal = 64%
3	HALLMARK_CHOLESTEROL_HOMEOSTASIS	74	−0.35663486	−1.3603169	0.03846154	0.11938669	0.047	3913	tags = 43%, list = 20%, signal = 54%
4	HALLMARK_INTERFERON_GAMMA_RESPONSE	200	−0.25014699	−1.24814	0	0.19125329	0.092	3746	tags = 37%, list = 19%, signal = 45%
5	HALLMARK_FATTY_ACID_METABOLISM	156	−0.2812873	−1.2210402	0	0.19619812	0.109	2894	tags = 26%, list = 15%, signal = 30%

### Identification of hub genes associated with *SLC25A21* expression

**FIGURE 5:**
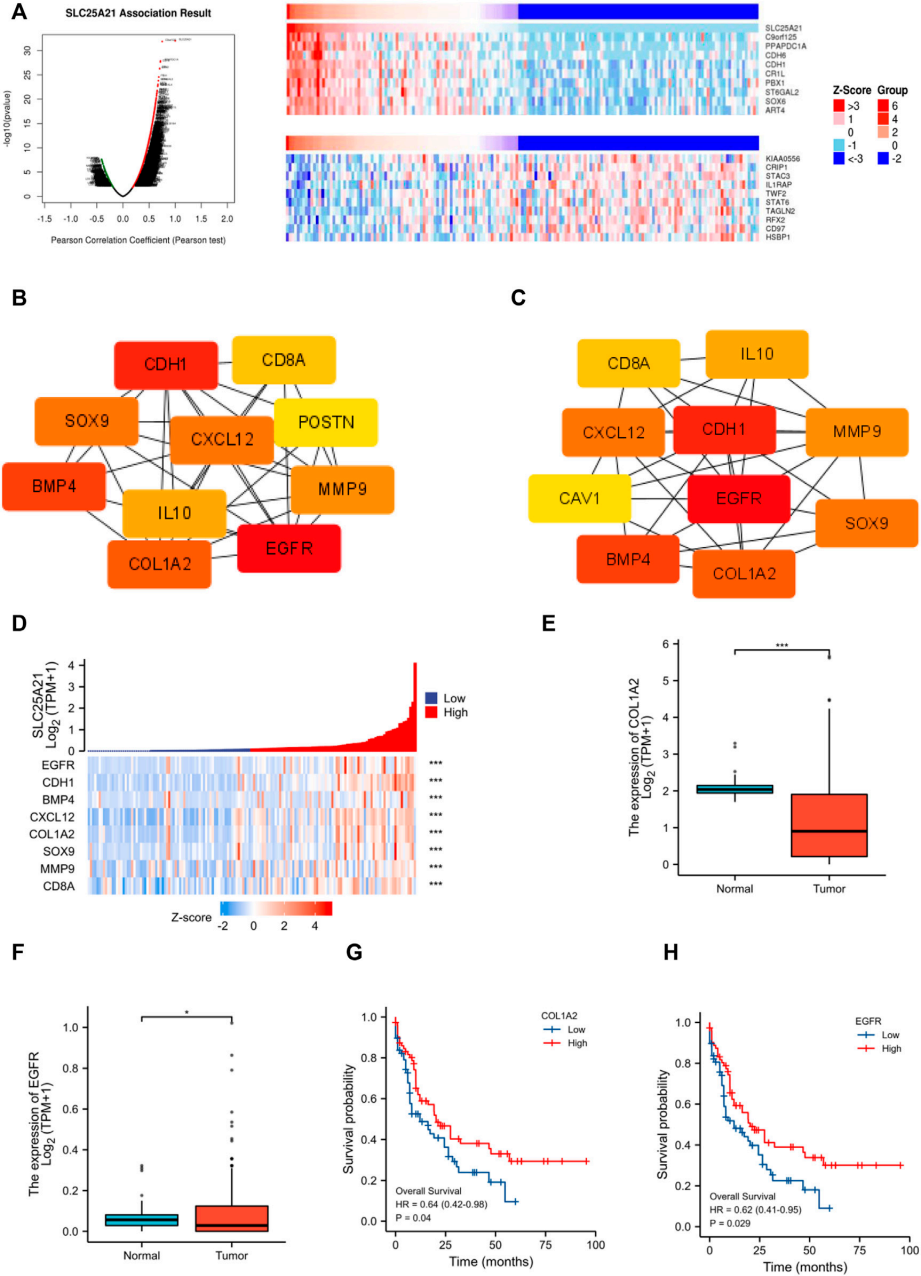
PPI network construction and clinical significance of hub genes. **(A)** Coexpression analysis of SLC25A21 in the TCGA-LAML dataset. The top 10 positively/negatively correlated genes are displayed. **(B–C)** The top 15 hub genes were selected on the basis of **(B)** MNC and **(C)** degree. **(D)** The association of SLC25A21 with eight hub genes (EGFR, CDH1, BMP4, CXCL12, COL1A2, SOX9, MMP9, and CD8A). **(E)** Expression levels of COL1A2 in AML patients (*n* = 132) and normal participants (*n* = 70). **(F)** Expression levels of EGFR in AML patients (*n* = 132) and normal participants (*n* = 70). **(G)** The difference in OS between patients with high and low COL1A2 expression levels shown by Kaplan-Meier curves. **(H)** The difference in OS between patients with high and low EGFR expression levels shown by Kaplan-Meier curves. (*, *p* < 0.05; **, *p* < 0.01; ***, *p* < 0.001).

Next, we constructed and analysed the PPI network and coexpression modules. As indicated in Figure 5A, most genes in AML were positively correlated with the expression of *SLC25A21*. A DEG-related PPI network was constructed to determine hub genes. The top 10 hub genes were identified by the MNC and Degree methods by using the cytoHubba plug-in of Cytoscape (Figures 5B,C). Furthermore, we observed eight shared hub genes (*EGFR*, *CDH1*, *CXCL12*, *CD8A*, *MMP9*, *SOX9*, *BMP4*, and *COL1A2*) between the above two gene lists. In addition, we detected the associations between *SLC25A21* and hub genes. The results showed that *SLC25A21* had significant correlations with *EGFR* (*p* value <0.001, correlation coefficient: 0.569), *CDH1* (*p* value <0.001, correlation coefficient: 0.709), *CXCL12* (*p* value <0.001, correlation coefficient: 0.590), *CD8A* (*p* value <0.001, correlation coefficient: 0.441), *MMP9* (*p* value <0.001, correlation coefficient: 0.295), *SOX9* (*p* value <0.001, correlation coefficient: 0.438), *BMP4* (*p* value <0.001, correlation coefficient: 0.383), and *COL1A2* (*p* value <0.001, correlation coefficient: 0.538) (Figure 5D). Finally, we examined the relationship between the levels of hub genes and prognosis and found that only *EGFR* and *COL1A2* were positively correlated with *SLC25A21* and linked to poor clinical outcomes in patients with AML (Figures 5E–H).

### Correlation analysis of *SLC25A21* and immune cells or immune checkpoint molecules

**FIGURE 6:**
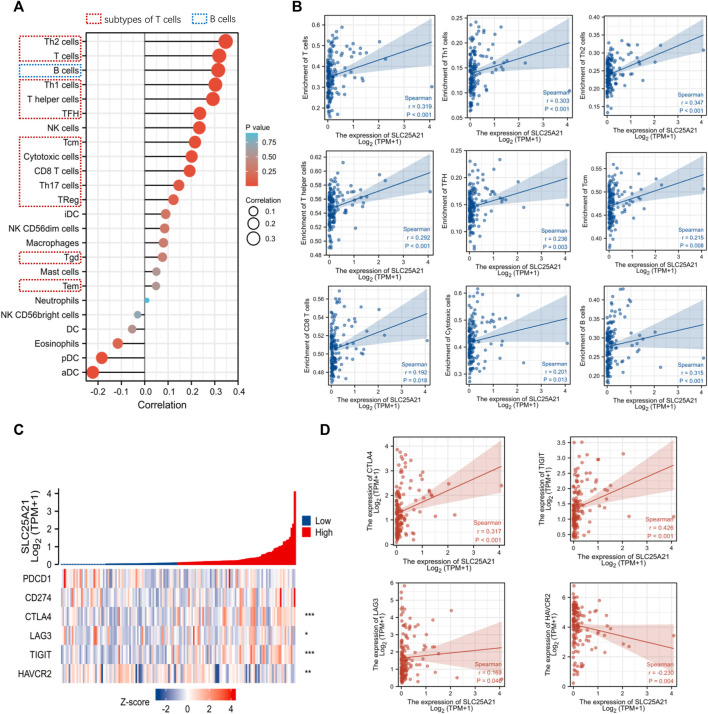
Correlation analysis between the level of SLC25A21 expression and immune cell infiltration or immune checkpoint molecules. **(A)** The relative contents of 24 kinds of immune cells in AML. **(B)** Spearman’s correlations were used to quantify the correlation of SLC25A21 expression with the number of infiltrating level B cells and subtypes of T-cells. **(C)** The association of SLC25A21 with five immune checkpoint molecules (PDCD1, CD274, CTLA-4, LAG-3, TIGIT, and HAVCR2). **(D)** Spearman’s correlation was used to quantify the correlation of SLC25A21 expression with immune checkpoint molecules (r is Spearman’s correlation coefficient).

Tumor infiltrating lymphocytes affect the survival of patients with various cancers. Therefore, 24 kinds of infiltrating immune cells were evaluated to describe the association between the levels of *SLC25A21* expression and immune infiltration in AML. The results showed that the expression level of *SLC25A21* had an obvious positive correlation with the numbers of infiltrating B cells, T-cells, Th1 cells, Th2 cells, T helper cells, Tfh cells, CD8 T-cells, cytotoxic cells and Tcm cells (Figure 6A). The details of the quantitative analysis with Spearman’s correlation coefficient are shown in Figure 6B.

Furthermore, we clarified the relationship between *SLC25A21* and immune checkpoint (*PDCD1*, *CD274*, *CTLA4*, *LAG-3*, *TIGIT*, and *HAVCR2*) expression. In our study, *SLC25A21* was significantly correlated with *CTLA4*, *LAG3*, *TIGIT*, *CD274*, and *TIGIT*. Details of the correlation analysis are shown in Figures 6C,D.

### Drug sensitivity analysis

**FIGURE 7:**
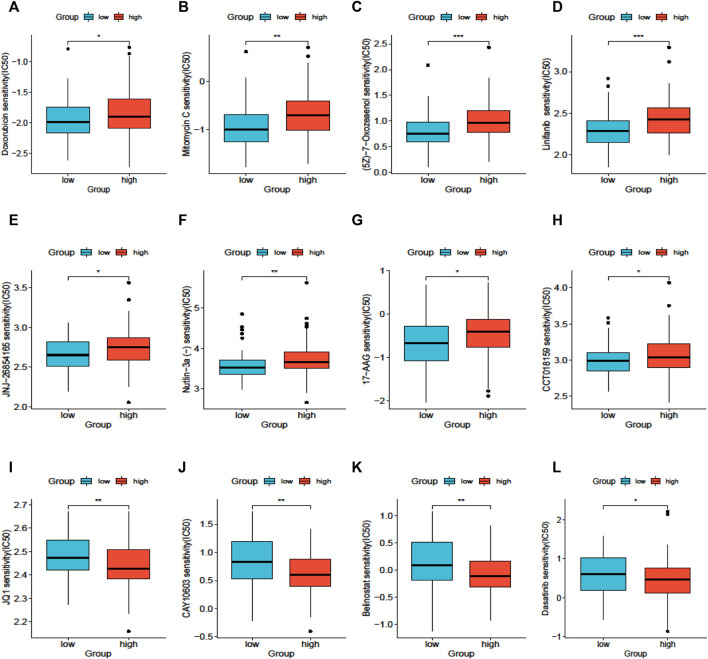
Drug sensitivity analysis based on SLC25A21. **(A)**, Doxorubicin. **(B)**, Mitomycin **(C)**, 7-Oxozeaenol. **(D)**, Linifanib. **(E)**, JNJ-26854,165. **(F)**, Niutlin-3a. **(G)**, 17-AAG. **(H)**, CCT018159. **(I)**, JQ1. **(J)**, CAY10603. **(K)**, Belinostat. **(L)**, Dasatinib.

The results of drug sensitivity analysis for the high- and low-*SLC25A21* groups showed that the *SLC25A21* low expression group may be more sensitive to cell cycle inhibitors (doxorubicin and mitomycin C), vascular endothelial growth factor receptor (VEGFR) tyrosine kinase inhibitors (linifanib and 7-oxozeaenol), p53 activators (JNJ-26854165 and Nutlin-3a), and heat shock protein 90 (HsP90) inhibitors (CCT018159 and 17-AAG) but resistant to histone deacetylase (HDAC) inhibitors (JQ1, CAY10603, and belinostat) and tyrosine kinase inhibitors (dasatinib). These results indicated that *SLC25A21* has a significant correlation with chemotherapy and targeted therapy regimens for AML (Figures 7A–L).

## Discussion

AML is a highly heterogeneous disease with various cytogenetic and genetic alterations. Genetic abnormalities are not only the pathogenic basis of AML, but they have important treatment and prognostic implications. In this study, we screened transcriptome data for AML in public databases to discover novel molecular biomarkers with a potential impact on prognosis and/or therapeutic response. We identified DEGs between AML patients and healthy donors in two independent cohorts. Therefore, a list of 14 AML-specific genes was obtained, including *IL1R2*, *MMP8*, *FGF13*, *SLC25A21*, etc.

(Le Sommer et al., 2018; Nobrega-Pereira et al., 2018) *SLC25A21* SLC25A21 is a 2-oxoglutarate transporter embedded in the mitochondrial inner membrane and, in some cases, organelle membranes. The expression of human *SLC25A21* has a wide distribution with very little variation between tissues. A recent study revealed that *SLC25A21* suppresses cell growth and plays a pathogenic role on bladder cancer (Wang et al., 2021). Recent studies have shown that metabolic molecules are dysregulated in AML cells and play key roles in leukaemogenesis, contributing to chemoresistance and disease relapse (Le Sommer et al., 2018; Nobrega-Pereira et al., 2018). Targeting cell metabolism is now considered a viable therapeutic strategy for AML. Therefore, we focused on the metabolism-related gene *SLC25A21* for further studies.

Given the above, we first explored the association of *SLC25A21* gene expression levels with main clinical features in TCGA-LAML cohorts of AML patients. *SLC25A21* We found that *SLC25A21* was significantly downregulated in AML patients. As expected, a low level of *SLC25A21* was associated with higher WBC counts, higher BM and PB blast abundance and poor prognosis. Thus, we speculated that abnormally low expression of *SLC25A21* plays an unfavourable role in promoting AML cell proliferation and survival while preventing leukaemic cell differentiation.

Hence, we explored the possible molecular mechanism underlying this association by using a bioinformatics approach. The enriched GO terms and KEGG pathways were mainly involved in growth factor binding, collagen-containing extracellular matrix, extracellular structure organization, the PI3K-Akt signalling pathway and the Wnt signalling pathway. Concurrently, GSEA showed enrichment of epithelial-to-mesenchymal transition, the KRAS signalling pathway, oxidative phosphorylation, DNA repair and the reactive oxygen species (ROS) pathway.

Mitochondria are the primary intracellular source of ROS and play important roles in aerobic metabolism and oxidative phosphorylation. Thus, dysregulation of mitochondrial metabolism is closely related to the development and progression of haematopoietic malignancies (Basak and Banerjee, 2015; Porporato et al., 2018). As a carrier embedded in mitochondria, overexpression of *SCL25A21* resulted in efflux of α-KG from mitochondria, leading to upregulation of ROS accumulation, which in turn induced mitochondrial apoptosis (Wang et al., 2021). Moreover, increased ROS levels drive a cycle of genomic instability. Leading to DNA double-strand breaks (DSBs) and altered DNA repair. The accumulation of intracellular ROS can promote tumour proliferation, but excessive accumulation of ROS can lead to cell apoptosis (Trotta et al., 2017; Aggarwal et al., 2019). Recent studies have revealed that the majority of functionally defined leukaemia stem cells (LSCs) are functionally characterized by relatively low levels of ROS. Meanwhile, several EMT-related genes conferring properties of “stemness” were strongly associated with shorter OS in AML patients (Stavropoulou et al., 2016; Carmichael et al., 2020; Almotiri et al., 2021). More importantly, the PI3K and KRAS signalling pathways play important roles in the proliferation and differentiation of haematopoietic cells (Crespo and Leon, 2000; Martelli et al., 2009; Nepstad et al., 2018). Thus, we speculated that the pathological mechanism of *SLC25A21* may be related to these signalling pathways.

Furthermore, through a series of rigorous screens, two hub genes (*EGFR* and *COL1A2*) that could accurately predict the prognosis of AML were found. It has been reported that dysregulation of *EGFR* can lead to the development of malignancy (Cheng et al., 2011; Singh et al., 2016). *EGFR* repairs the DNA of HSCs by activating DNA-dependent protein kinase catalytic subunit (DNA-PKcs), leading to the regeneration of normal haematopoietic cells. Experimental studies have shown that deletion of *EGFR* in progenitor cells results in reduced DNA-PKcs activity, thus reducing the ability of the cells to regenerate normal HSCs (Fang et al., 2020). In AML, we speculate that EGFR may play the same role to inhibit normal haematopoiesis, resulting in a shorter survival time for patients with low EGFR expression. *COL1A2* has also been implicated in gastric cancer, colorectal cancer, prostate cancer, pancreatic cancer (Yu et al., 2018; Wu et al., 2019; Nie et al., 2020; Liu et al., 2022), and so on. Moreover, *COL1A2* has been identified as a hub gene in *FLT3*-mutated AML (Chen et al., 2020b). Although *SLC25A21, EGFR*, and *COL1A2* are linked to tumour-associated signalling pathways, the precise mechanisms of this synergy remain unclear. Further in-depth studies are needed to address this issue in more detail.

Additionally, metabolic molecular abnormalities may facilitate AML cell escape and immune detection and severely reduce the efficacy of immunotherapy (Mougiakakos, 2019). Several studies have shown that the level of immune infiltration and immune evasion mechanisms of AML cells determine their immune evasion ability (Rosenthal et al., 2019). Therefore, we investigated the relationship between *SLC25A21* expression and immune infiltration levels in AML patients and found that with downregulation of *SLC25A21*, the infiltration levels of various T-cells and B cells were greatly decreased. Next, we also showed that *SLC25A21* expression had a positive correlation with some immune checkpoint genes (CTLA-4, LAG3, TIGIT, and HAVCR2), which serve as activation markers of T-cells and affect antitumor immunity. We speculate that this may be because the number of infiltrating immune cells is significantly reduced, resulting in decreased expression of molecular markers of T-cell activation. These results suggest that *SLC25A21* may lead to immune escape in AML. This observation may provide a framework to guide further investigation of *SLC25A21* in clinical and basic science research.

Last, the ultimate objective of our research is to provide clinicians with guidelines to choose the appropriate therapeutic regimens for each AML patient. With the development of next-generation sequencing technology, several genetic aberrations have been found to contribute to drug resistance in AML (Gollner et al., 2017; Hou et al., 2017; Nechiporuk et al., 2019). In this study, we analysed the correlation between *SLC25A21* and drug resistance in AML. Patients with low *SLC25A21* expression levels were sensitive to doxorubicin, mitomycin, lapatinib, midostaurin, sorafenib, linifanib, Nutlin-3a, 17-AAG, 5-fluorouracil, 7-oxozeaenol, JNJ-26854165, CCT018159, bleomycin, and FH535 but resistant to JQ1, CUDC-101, dasatinib, and GNF-2. These results indicate that downregulation of *SLC25A21* may promote sensitivity to doxorubicin, the cornerstone regimen for AML. These results suggest that while *SLC25A21* affects prognosis in AML, patients with low expression of *SLC25A21* may still benefit from traditional chemotherapy regimens.

However, our study has several limitations. First, we explored the mutational frequency of *SLC25A21* in 6 independent AML studies (*n* = 2,177) and found a frequency of approximately 0.1%–0.2% (Supplementary Figure S2). In addition, we observed enrichment of transcriptional regulation pathways in GO analysis. Therefore, the upstream transcriptional regulatory mechanism of *SLC25A21* remains to be uncovered. Second, we evaluated the diagnostic value and drug sensitivity of *SLC25A21* by using public resources. However, we have not yet tested some new clinically emerging targeted drugs, such as venetoclax, due to the limitations of the training database. Last, all associations between *SCL25A21* and AML-associated immune molecules lack functional validation and detection of the potential cellular and molecular mechanisms. Future studies will build on these points with a view toward providing new options for precision medicine approaches and improving the treatment of AML patients.

## Conclusion

Taken together, our preliminary findings showed that low expression levels of the metabolism-related gene *SLC25A21* had an unfavourable effect on the overall survival of AML patients and may be correlated with immune escape. A low level of *SLC25A21* could be an independent predictor of poor prognostic for AML patients. This discovery could promote the development of novel targeted drugs and provide therapeutic options for personalized therapy.

## Data Availability

The datasets presented in this study can be found in the GEO,TCGA and GETx repositories: https://www.ncbi.nlm.nih.gov/geo/; https://portal.gdc.cancer.gov/projects/TCGA-LAML; https://xenabrowser.net/datapages/. The accession numbers can be found in the article.

## Supplementary material

SUPPLEMENTARY FIGURE S1
**(A)**, SLC25A21 expression was not associated with cytogenetics in AML. **(B)**, SLC25A21 expression was not associated with French–American–British (FAB) classifications.

SUPPLEMENTARY FIGURE S2
**(A)**, Mutation frequency of *SLC25A21* in leukaemia.

SUPPLEMENTARY TABLE S1 Univariate and multivariate Cox regression analysis.

SUPPLEMENTARY TABLE S2 GO enrichment and KEGG pathway analysis.

SUPPLEMENTARY TABLE S3 Patient sample information.

SUPPLEMENTARY TABLE S4 List of primer sequences.
