# Supplementary figures and images for: Low expression of the metabolism-related gene SLC25A21 predicts unfavourable prognosis in patients with acute myeloid leukaemia
Source: Front Genet. 2022 Sep 30;13:970316. doi: 10.3389/fgene.2022.970316 (PMC9562002; doi:10.3389/fgene.2022.970316)

A

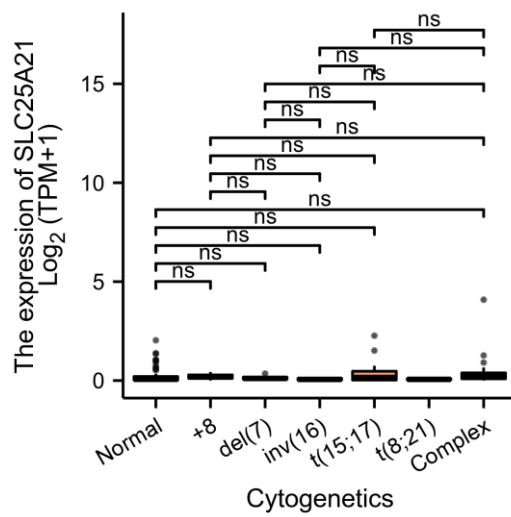

B

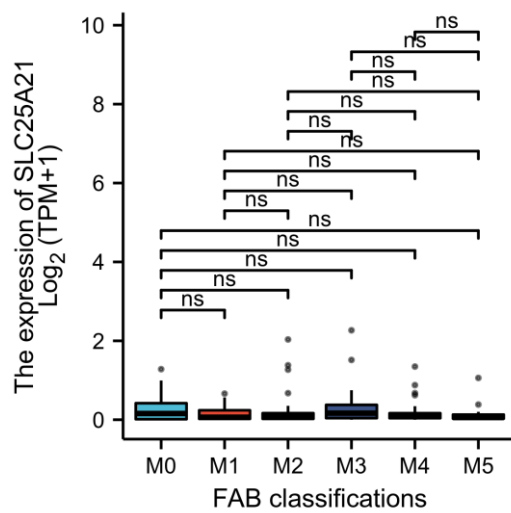

Supplement: Supplementary file 5 [file DataSheet1.PDF]
