# Supplementary material for: Low expression of the metabolism-related gene SLC25A21 predicts unfavourable prognosis in patients with acute myeloid leukaemia
Source: Front Genet. 2022 Sep 30;13:970316. doi: 10.3389/fgene.2022.970316 (PMC9562002; doi:10.3389/fgene.2022.970316)
Supplement: Supplementary file 1 [file DataSheet2.PDF]

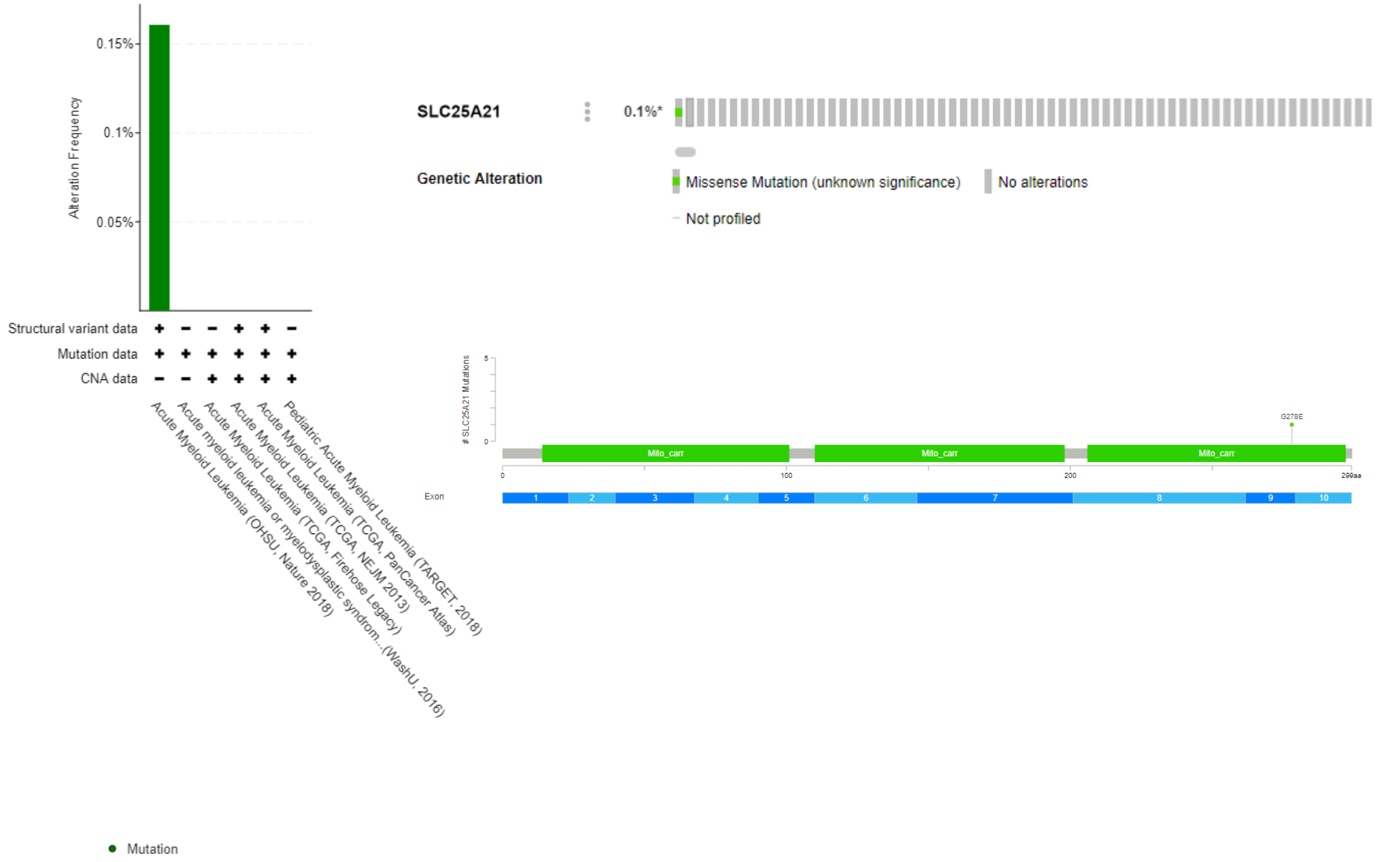
Alteration Frequency
0.15%
0.1%
0.05%
Structural variant data
Mutation data
CNA data
Acute Myeloid Leukemia (OHSU, Nature 2018)
Acute myeloid leukemia or myelodysplastic syndrom...(WashU, 2016)
Acute Myeloid Leukemia (TCGA, Firehose Legacy)
Acute Myeloid Leukemia (TCGA, NEJM 2013)
Acute Myeloid Leukemia (TCGA, PanCancer Atlas)
Pediatric Acute Myeloid Leukemia (TARGET, 2018)
Mutation
SLC25A21
0.1%*
Genetic Alteration
Missense Mutation (unknown significance)
No alterations
Not profiled
SLC25A21 Mutations
G278E
Mito_carr
Mito_carr
Mito_carr
0
100
200
299aa
Exon
1
2
3
4
5
6
7
8
9
10
